# Between distance and duty: emotional labor in transnational fatherhood among Pakistani migrants in Italy

**DOI:** 10.3389/fsoc.2025.1643074

**Published:** 2025-09-01

**Authors:** Syed Imran Haider

**Affiliations:** ^1^Marie Sklodowska Curie Research Fellow, Ca Foscari University of Venice, Venice, Italy; ^2^Allama Iqbal Open University, Islamabad, Pakistan

**Keywords:** migration, migration and society, fatherhood, transnational fatherhood, parenting, emotional labor, love and care, family studies

## Abstract

**Introduction:**

Transnational fatherhood presents unique emotional and caregiving challenges, especially for migrant fathers navigating life across borders. Parenthood studies often focused motherhood in the context of transnational families and men are usually seen from the perspectives of labor laws, immigration policies and economy. This phenomenological study explores the lived experiences of Pakistani migrant fathers in Italy, examining how they balance emotional connections and caregiving responsibilities with economic imperatives being away from their families.

**Methods:**

The study adopted phenomenological approach and 30 in depth interviews were conducted with purposively selected Pakistani migrants residing for at least 5 years in Bergamo and Naples, Italy.

**Results and discussion:**

The research highlights six key themes such as (1). Emotional pain of physical separation, (2). Helplessness during family illness/crisis, (3). Emotional impact on special occasions, (4). Fear of weakening family bonds, (5). Guilt, Stress, and Identity conflict, and (6). Coping through faith and hope for relieving emotional sufferings and challenging situations. The results explicate that emotional toll of long-distance fatherhood included feelings of loneliness, guilt and sadness and fathering role was exercised through engaging in digital caregiving and regular communication through audio/video calls using digital technology tools. Extreme feelings of stress and anxiety were reported on the specific occasions including illness or death of a family members and special events like birthdays, marriages or religious festivals. The stories of transnational fathers highlighted their turning to religion and accepting is as fate as vital coping mechanism to relieve emotional strain. The study highlights the need for psychosocial initiatives and engaging activities to reduce the feelings of loneliness.

## Introduction

Transnational fatherhood, particularly within the context of labor migration, introduces a complex set of emotional, relational, and caregiving challenges (Lee and Macías-Ayala, 2025). For Pakistani migrant fathers in Italy, the experience is marked by the constant tension between economic provisioning and emotional caregiving across physical and cultural distances (del Carmen Ernstberger and Adaawen, 2023; Katsampa et al., 2025; Mukhtar, 2020). Migration transforms traditional understandings of fatherhood rooted in physical co-presence, authority, and direct supervision into new, often improvised, forms of remote parenting (Baldassar, 2008; Parreñas, 2005). These fathers must navigate the emotional costs of separation, including loneliness, anxiety, and guilt, while simultaneously striving to fulfill the provider role expected of them by their families and communities (Horton, 2009; Parke and Cookston, 2021). This emotional labor is often rendered invisible within migration discourses that prioritize economic contributions over caregiving roles, thereby obscuring the profound psychological toll fathers endure (Coe, 2011; Kumari and Singh, 2025; Mpaata, 2021).

The emotional dissonance experienced by migrant fathers is not only internal but also relational (del Carmen Ernstberger and Adaawen, 2023; Lan, 2023). Left-behind children may struggle to bond with or recognize the authority of an absent father, especially when reunification occurs after years of separation (Parreñas, 2005; Poeze, 2019). Fathers often report feelings of rejection and emotional estrangement upon return, as family members have developed new dynamics in their absence. The reconstitution of family roles in the host or home country particularly the shift in caregiving responsibilities to mothers or extended kin further complicates paternal re-engagement (Hoang and Yeoh, 2012; Salazar Parreñas, 2008). For Pakistani fathers, who often derive their social identity from religious and cultural ideals of fatherhood rooted in presence and protection, these disruptions can result in deep emotional insecurity and a perceived loss of authority (Charsley, 2005; Jeong et al., 2018). Additionally, precarious labor markets in Italy, marked by racialized job segregation and limited access to social services, add another layer of vulnerability to their emotional and caregiving capacities (Ba', 2024; Coe, 2011; Molina, 2015).

Despite these constraints, transnational fathers are not passive victims of structural limitations but active agents who develop diverse caregiving strategies. Digital technologies such as WhatsApp, video calls, and social media have become indispensable tools for migrant fathers to remain emotionally and morally present in their children's live (Cogo, 2017; Nedelcu and Wyss, 2016). These technologies facilitate virtual co-presence, allowing fathers to participate in key moments, assist with homework, provide religious instruction, and offer emotional reassurance (Baldassar, 2008; Larrinaga-Bidegain et al., 2024; Madianou and Miller, 2011). Many engage in symbolic caregiving through personalized remittances, gifts, or messages, using these gestures to reinforce their affective ties and cultural values (Cabalquinto, 2020; Molina, 2015; Poeze, 2019). Nevertheless, digital connectedness does not always guarantee emotional intimacy; it may sometimes highlight the distance rather than bridge it, especially when technological literacy, time zones, or labor demands hinder communication (Chen, 2020; Gonzalez and Katz, 2016; Wang and Lim, 2020; Wilding, 2006).

A critical review of the literature reveals important theoretical and methodological gaps. Majority of transnational parenting studies have focused on maternal migration or examined fatherhood primarily in African, Latin American, or Southeast Asian contexts (Bonizzoni and Boccagni, 2013; Caarls et al., 2018; Parreñas, 2005; Pineros-Leano et al., 2021; Raffaeta, 2016). South Asian fathers, particularly from Pakistan, remain significantly underrepresented, especially in Southern European countries like Italy, where distinct migration patterns and sociopolitical dynamics shape caregiving experiences (Tosi and Impicciatore, 2022). Furthermore, the prevailing analytical frameworks often treat fathers as economic actors, neglecting the emotional and symbolic dimensions of their caregiving practices. There is also a lack of ethnographic engagement with fathers' own perspectives, as many studies rely on the accounts of left-behind family members or focus on policy-level analyses (Doucet, 2020; Yeoh and Lam, 2007). This omission limits the field's capacity to understand how migrant fathers construct, negotiate, and reimagine fatherhood in transnational spaces. Statistics also highlight the need as, during year 2021, 2.3 million new immigrants, mostly men, entered EU states and 5.4 migrants per 1,000 inhabitants are in Italy 23. Amongst 1 million Pakistanis in Europe, 58% are males and out of 124,800 documented Pakistani migrants in Italy, men constitute 72%. Pakistanis are currently the second largest diaspora of any nationality in Italy. Additionally, a continuous inflow from South Asia through legal and illegal migration highlight the importance of working with this group.

This study focuses on Pakistani migrant fathers in Bergamo (Lombardy) and Naples (Campania) due to the significant concentration of Pakistani diaspora in these regions, offering a robust sampling frame (Ambrosini, 2017). Furthermore, selecting sites across Italy's economically diverse North (Lombardy) and South (Campania) allows for a nuanced exploration of how contrasting labor markets, social integration dynamics, and regional migration histories shape fathers' transnational experiences and emotional labor (Perrotta and Nocifora, 2019; Sciortino, 2011). It examines how these fathers navigate the emotional strain of family separation, develop cross-border caregiving strategies, and confront shifting gender norms in both home and host societies. By centering fathers' voices through qualitative interviews, the study provides a grounded and nuanced account of transnational fatherhood that challenges conventional binaries of provider vs. caregiver. It contributes to broader discussions on masculinity, migration, and emotional labor. In doing so, this research underscores the importance of culturally sensitive policy interventions that acknowledge both the material and emotional dimensions of transnational caregiving.

## Objectives

Following were the main objectives of the study:

1. To unpack the forms and emotional costs of emotional labor performed by Pakistani migrant fathers in Italy as they strive to maintain cross-border familial ties and fulfill their paternal duties.
2. To analyze how digital technologies mediate and transform the emotional labor of transnational fatherhood, enabling fathers to navigate physical distance while confronting new relational dynamics and challenges to traditional paternal authority.
3. To explore how the intersection of cultural ideals of Pakistani fatherhood, host country structural realities, and family dynamics shapes fathers' emotional experiences and the coping strategies they develop in their transnational lives.

## Theoretical framework

This study employs Arlie Russell Hochschild's concept of emotional labor as its core theoretical lens, extending it from public service work to the intimate, transnational family sphere (Hochschild, 1983). For Pakistani migrant fathers in Italy, emotional labor involves the active management of feelings to maintain affective ties and paternal roles across physical distance, often entailing significant psychological costs. Integrating this with theories of transnationalism and masculinity, the framework explores how fathers navigate the tension between economic provisioning and their emotional presence, challenging traditional notions of fatherhood and revealing how they reimagine their roles through cross-border emotional work. This approach moves beyond viewing migrant fathers solely as economic actors, offering a nuanced understanding of their complex caregiving practices and the emotional toll they endure.

## Methodology

The study employs phenomenological approach to explore the emotional and caregiving experiences of Pakistani transnational fathers living in Italy. This approach seeks to uncover the essence of experiences by describing phenomena as they are lived and perceived by participants (Creswell and Poth, 2016; Dodgson, 2023). In the context of transnational fatherhood, this approach is especially relevant because it allows for deep engagement with subjective emotional realities, including feelings of loss, guilt, longing, and resilience, which are often underexplored in migration research that tends to focus more on structural or economic dimensions (Collinson, 2009; Penninx, 2005).

### Setting

This study was conducted in Bergamo and Naples, two Italian cities that host substantial communities of Pakistani migrant workers (Mukhtar, 2020).

### Sample and recruitment

The participants of this study were Pakistani migrant fathers residing in Italy for at least 5 years, who had children living in Pakistan. A total of 30 participants were selected through purposive and snowball sampling techniques (Keshavarz et al., 2018; Sultan et al., 2020), ensuring representation from varied educational backgrounds, occupations, lengths of stay in Italy, and legal statuses. Fathers were approached at locations that were comfortable and accessible for them.

### Data collection

Data were collected through in-depth interviews, which are standard tools in phenomenological research due to their open-ended nature and depth (Seidman, 2006; Smith et al., 2014). Interviews were conducted in Urdu and Punjabi to enable participants to express their feelings in culturally and emotionally resonant terms. Each interview lasted between 60 and 90 min and was conducted in a setting chosen by the participants to ensure comfort and openness. All interviews were audio-recorded with participants' consent, transcribed verbatim, and then translated into English by bilingual researchers to ensure cultural and linguistic accuracy.

### Data analysis and trustworthiness

The transcribed data were analyzed using thematic analysis, guided by Clarke and Braun (2017) six-step framework. To ensure analytic rigor, two independent researchers reviewed the data and collaboratively developed a codebook. Data analysis was further supported by MAXQDA qualitative analysis software, which allowed for efficient data organization and theme tracking.

### Ethical considerations

Ethical approval was obtained from the Ethics committee of Ca Foscari University of Venice. All participants were provided with detailed information about the research objectives, interview process, and confidentiality measures. Verbal informed consent was obtained for both participation and audio recording. Participants' identities were anonymized, and all data were stored securely. Care was taken to respect participants' dignity, emotional comfort, and freedom to withdraw at any stage.

## Results and discussion

Most of the participants of this study had been residing in Italy for extended periods, with their duration of stay ranging from 6 to 14 years, and an average of 8–9 years. Most interviewees originated from the Punjab province of Pakistan, particularly from semi-urban or rural backgrounds, where traditional family structures and expectations around masculine responsibility are deeply embedded. In Italy, their livelihoods spanned a range of low-wage, labor intensive sectors including factory work, warehouse operations, restaurant kitchens, food delivery, and retail sales in small shops. Many worked irregular or rotating shifts. Many participants reported daily working hours between 8 and 12 working hours with frequent transitions between morning, evening, and night shifts. Housing arrangements typically involved communal living, with 4–8 men sharing modest rented apartments or shared rooms to minimize expenses. It was observed that the shared living also facilitated informal networks of emotional support and mutual care, particularly among fellow migrants from Pakistan.

Despite their modest earnings, participants routinely sent remittances to support not only their nuclear families but also extended kin in Pakistan, including parents, siblings, and in-laws. Many reported being the sole breadwinners for multigenerational households. Their family structures in Pakistan often included wives and two to four children, and in some cases co-residing elders. This transnational responsibility created a persistent emotional burden, as they strived to maintain fatherhood and familial presence across distance using digital tools, all while navigating precarious working conditions and social isolation in Italy.

Based on the study findings, six thematic areas emerged from the narratives of the transnational Pakistani fathers living in Italy including (1). Emotional pain of physical separation, (2). Helplessness during family illness/crisis, (3). Emotional impact on special occasions, (4). Fear of weakening family bonds, (5). Guilt, Stress, and Identity conflict, and (6). Coping through faith and hope. These themes encapsulate the multifaceted emotional and psychological experiences of individuals living away from their families.

## Emotional pain of physical separation

The emotional burden experienced by Pakistani transnational fathers living in Italy was profoundly shaped by the pain of physical separation from their children and families. This pain, often internalized and masked by external composure, reflected a deep emotional struggle that defined their everyday lives. One father in Bergamo shared,

“*Every morning, I wake up with a smile for the world, but deep inside, I carry a constant ache—I haven't hugged my daughter in over two years*,”

These sentiments underscore the intense affective cost of transnational caregiving, where geographic distance erodes the immediate intimacy of parenting (Parreñas, 2005). Such narratives reveal the duality of outward resilience and internal suffering, a phenomenon widely observed among migrant fathers who must reconcile their roles as distant providers with their emotional longing for familial closeness (Carling et al., 2012; Madianou and Miller, 2011). Migrant parents often perform emotional labor by projecting strength and financial provision while simultaneously battling internal emotional voids (Fresnoza-Flot, 2009). This dissonance between public performance and private pain reflects the structural constraints of migration that force men into redefined paternal roles, often reduced to economic providers rather than emotional caregivers (Madianou and Miller, 2011). Additionally, the prolonged absence may lead to feelings of guilt, emotional disconnection, and the fear of being forgotten in their children's lives (Carling et al., 2012; Lee and Macías-Ayala, 2025). Another father, based in Naples, recalled,

“*I held my son for the first time when he was five months old during a visit to Pakistan. That memory is what keeps me going, but it also breaks me every night*”

This highlights how fragmented moments of physical closeness become both a source of solace and grief. These transient reunions are not merely emotional anchors but potent reminders of lost time, disrupted intimacy, and the emotional labor of long-distance fatherhood. For many migrant fathers, caregiving is not merely financial but deeply emotional, rooted in presence, touch, and daily engagement with their children's growth and wellbeing (Carranza, 2022; Haagsman and Mazzucato, 2014; Kay, 2009). These fathers often navigate a paradox of caregiving from a distance, where their roles are performed through remittances and virtual communication, yet remain insufficient to fulfill the emotional needs of both parent and child (Poeze, 2019; Salazar Parreñas, 2008). The memory of brief, precious moments like holding a child for the first time becomes both a sustaining force and a psychological wound, symbolizing what has been gained through migration and what has been irretrievably lost. Moreover, this emotional pain is not just personal but culturally mediated, as traditional South Asian notions of fatherhood emphasize close paternal involvement and moral guidance, making the absence all the more emotionally taxing (Kay, 2009; Nasar et al., 2025). As such, the emotional cost of migration, especially for fathers, extends beyond economic sacrifice to a profound disruption in the paternal identity and sense of belonging within the family. Similarly, a Pakistani father in Bergamo reflected,

“*I remember the weight of my daughter in my arms when I visited, now she must have grown so much. And I wasn't there to see it*”

It highlights the sorrow of missing developmental milestones, which deepens the emotional chasm created by migration. These missed moments such as first words, first steps, birthdays etc. become emblematic of the intangible losses these migrant fathers endure, evoking a sense of time slipping away without the ability to participate meaningfully in their children's lives. Such experiences highlight how transnational fatherhood is not simply a reconfiguration of roles but a fragmentation of paternal identity, where the physical absence compromises emotional bonds despite sustained financial support or virtual communication (Shaw, 2019). Fathers often grapple with an internal conflict between the ideal of involved fatherhood and the practical realities of distance, which can lead to feelings of failure and emotional alienation (Moila, 2023).

It also captures the enduring nature of emotional absence, how a single memory of physical closeness transforms into a painful reminder of everything that followed in their absence. While communication technologies offer ways to remain in contact, they cannot fully substitute the embodied experience of parenting, particularly in cultures like that of Pakistani families where direct, physical care and guidance by fathers remain central to notions of responsible fatherhood (Bacigalupe and Lambe, 2011; Marchetti-Mercer and Swartz, 2020). This emotional toll is exacerbated by gendered expectations and cultural pressures that discourage open expression of vulnerability among men, often forcing migrant fathers to suppress their grief in silence (McNeil et al., 2021). Thus, the pain of being physically distant from a growing child is not just about geography, but about the slow erosion of the father-child relationship, one marked by absence, longing, and a fractured paternal presence.

## Helplessness during family illness/crisis

This theme emerged as a powerful emotional burden among Pakistani migrant fathers in Italy, revealing a deep fracture in their perceived roles as protectors and caregivers. Not being able to be physically present to support or care for relatives led to significant emotional turmoil and anxiety. This sense of powerlessness often exacerbated mental health challenges and contributed to chronic stress. One father from Naples expressed,

“*When my wife told me our son had dengue, I felt paralyzed. I couldn't even take him to the doctor myself. That helplessness kills me*”

This haunting expression captures the distressing reality of being unable to fulfill even the most basic paternal duty in times of medical emergencies. For migrant fathers, such moments are not only emotionally painful but profoundly disempowering. This emotional paralysis stems from a perceived loss of agency, as migrant fathers are reduced to distant observers rather than active participants in their children's wellbeing, a condition exacerbated by the structural constraints of immigration, legal status, and economic dependency (Adil et al., 2025; Fauk et al., 2024). The inability to respond physically during a child's illness undermines the traditional role of the father as protector and caregiver, central to many South Asian familial and cultural norms (Shari, 2009). Research has shown that the transnational arrangement often leads to fractured or delegated fatherhood, where the caregiving role is involuntarily outsourced to spouses or extended family members back home, while the father struggles with an emotional and symbolic disconnect (Tawodzera, 2021). Another father from Bergamo shared,

“*My youngest got hurt on the stairs, needed stitches. I saw the pictures later, but I wasn't there to hold him, to tell him it's okay. I felt like a useless father*”

This deeply personal account reflects how moments of crisis, even seemingly minor injuries, become emotionally charged turning points that amplify a migrant father's sense of regret, inadequacy, and emotional failure. In these instances, the inability to comfort or physically console one's child generates acute self-doubt and internal conflict. This emotional dissonance is especially pronounced in patriarchal cultural contexts, such as that of Pakistani families, where fatherhood is not only measured by economic provision but deeply intertwined with ideals of physical presence, authority, and emotional strength (Chohan and Habib, 2024; Kay, 2009; Shah, 2023). The inability to respond immediately to crises intensifies a father's sense of failure, as reflected in another father's painful question:

“*What kind of father am I if I can't protect my children when they cry or fall sick?*”

This rhetorical self-interrogation underlines the moral and emotional conflict inherent in transnational fatherhood, where financial support is often emphasized, yet emotional caregiving and protection remain out of reach. Despite remittances being critical for their families' survival, the migrant fathers often feel that their contributions are emotionally void when they are not physically present to comfort or care for their children (Bryceson, 2019). In moments of family crisis, the distance becomes a cruel reminder of what has been surrendered in pursuit of a better life.

## Emotional impact on special occasions

The emotional toll of transnational fatherhood is particularly acute during special occasions such as Eid, birthdays, and religious or cultural celebrations that are traditionally rich in family bonding and shared rituals. These moments reinforced the emotional cost of migration and long-term separation. As one father in Bergamo confided,

“*Eid is the hardest. Everyone here is happy, but I'm crying inside because I'm not with my family. I video call them, but it's not the same as sharing a hug after prayer*”

This sentiment illustrates how, despite the affordances of digital technology, emotional presence cannot be fully mediated through screens. Special occasions serve as temporal markers of absence, reinforcing the emotional distance between migrant fathers and their children. Also such absence increases depressive symptoms and feelings of loneliness (Silver, 2014). The symbolic and affective aspects of these moments such as physical embrace, shared meals, communal prayer cannot be replicated virtually, often leaving fathers with a profound sense of isolation and longing (Ngan and Chan, 2022). A father in Naples shared,

“*My daughter's birthday passed last month. I watched her blow the candles over a video call. She smiled, but my heart felt empty*”

This poignant reflection illustrates the emotional hollowness that can accompany virtual parenting within transnational families. While digital communication tools such as video calls offer an essential lifeline for maintaining contact, they often fall short in replicating the sensory and emotional richness of physical co-presence (Baldassar and Wilding, 2020; Kelly et al., 2021; Robbins et al., 2023). Moreover, fathers' sense of emotional emptiness speaks to the broader challenges of sustaining paternal identity at a distance. In many South Asian cultural frameworks, fatherhood is intertwined with physical presence during life events, offering guidance, blessing, and support (Kay, 2009). Failing to do so, regardless of financial contributions, can deeply affect a father's self-perception and mental wellbeing. Thus, even as modern technologies help bridge geographic separation, they simultaneously expose the limits of mediated intimacy in the context of transnational family life (Baldassar and Wilding, 2020; Marchetti-Mercer and Swartz, 2020). Another father noted,

“*On Eid, I wear new clothes, go for prayer, but I don't go to friends' gatherings anymore. It hurts too much*”

This withdrawal from social celebration reflects the emotional dissonance many migrant fathers experienced, highlighting an internal split between outward participation and inward grief. Scholars have argued that such emotional withdrawal may be a coping mechanism to avoid intensifying the pain of absence, but it also signals a deepening emotional estrangement not only from family but from host-country social life as well (Coe et al., 2011). These accounts underscore how holidays and milestones, normally occasions for joy, become sharp reminders of separation, sacrifice, and the emotional costs of transnational migration.

## Fear of weakening family bonds

The fear of weakening family bonds was a profound and recurring concern among migrant fathers, particularly when physical separation became prolonged and routine. As one father from Naples painfully observed,

“*Sometimes I fear they will grow up without knowing who I am. Just a man on a screen every week. That's not fatherhood*”

This deeply personal reflection articulates a core anxiety shared by many migrant fathers, the fear that emotional proximity cannot be sustained through digital contact alone. The concept of a “good father” extends beyond the traditional role of being merely a breadwinner; it also emphasizes the importance of close emotional proximity and active participation in the daily lives of one's child (Kay, 2009). Failing which, migrant fathers often face emotional rejection from their children upon returning home, as the long absence creates a sense of distance and unfamiliarity. At the same time, their wives frequently express frustration and fatigue from carrying the full weight of household responsibilities alone, describing themselves as “pseudo-widows” (Mummert, 2005). Another father from Bergamo echoed this sentiment:

“*I worry my children will become distant. They talk more with their mother. I'm becoming just the man who sends money*”

This reflects a common narrative in migrant literature, where fathers feel reduced to financial providers, roles that, while essential, do not fulfill the emotional and relational dimensions of fatherhood. On the other hand involved fatherhood makes men happier and healthier in their lives (Behson and Robbins, 2016; Van Bemmelen et al., 2015). The gendered division of caregiving, wherein mothers are physically present and emotionally engaged, often leads to fathers being sidelined in everyday parenting, reinforcing their peripheral status in the emotional life of the child (Kay, 2009; Mazzucato and Schans, 2011). Over time, this can cultivate a deep sense of paternal inadequacy and the fear that financial provision is being substituted for meaningful connection. A father from Naples expressed this conflict poignantly:

“*I left to build their future, but what if I'm losing them in the process?*”

Migration, while often pursued with the intent of securing better educational and economic outcomes for children, paradoxically places the parent-child bond at risk. This emotional dilemma mirrors what Dreby (2010) calls the “paradox of sacrifice,” where the pursuit of familial wellbeing through migration may undermine the very relational fabric it seeks to protect. Moreover, concerns around moral guidance are evident in another father's reflection:

“*My son is growing up. He needs guidance, but who will tell him right from wrong if I am not there to see what he does?*”

The absence of a father during formative years can create anxiety about children's behavioral development, especially in patriarchal cultural settings where the father's role is traditionally linked with discipline, mentorship, and value transmission (Jeong et al., 2023; Lakhani and Nadeem, 2017). These accounts underscore a key finding in transnational family research: that distance not only disrupts daily routines but also reshapes emotional hierarchies within the family, often to the detriment of the migrant father's perceived role and relational intimacy.

## Guilt, stress, and identity conflict

The emotional burden of transnational fatherhood is marked by an ongoing conflict between the roles of provider and parent, often leading to deep feelings of guilt, stress, and fractured identity. Such fathers often experience diminished mental health, guilt and feeling of being ill-equipped for parenthood (Barrett and Charlton, 2025). One father from Naples poignantly described,

“*Sometimes I ask myself, am I a provider or a father? Because I feel like I can't be both from here*”

This deeply introspective statement captures a central identity conflict experienced by migrant fathers, the painful dichotomy between fulfilling the economic responsibilities traditionally tied to masculinity and fatherhood, and providing the emotional presence and caregiving that define contemporary parental roles (Kay, 2009). This internal conflict reflects what (Souralová and Fialová 2017) identifies as the “provider-only” model of transnational fatherhood, where emotional and caregiving functions are outsourced to the mother or other caregivers in the home country (Tawodzera, 2021). A father in Bergamo shared,

“*I am working twelve hours a day in a warehouse, but at what cost? My son calls me 'uncle' sometimes by mistake*”

This moment is not merely a linguistic slip but a symbolic expression of emotional distance and identity loss. It reflects the kind of relational alienation that can occur when caregiving is outsourced to the remaining parent and the migrant father becomes a marginal figure in the child's daily life (Tawodzera, 2021). In many cultural contexts, including Pakistan's, fatherhood is intimately linked to visibility and authority within the family sphere (Bhamani, 2012; Jeong et al., 2018). Therefore, being misrecognized by one's own child strikes at the core of a father's social and emotional identity. This emotional dislocation is compounded by chronic guilt. Another father from Naples confessed,

“*When I see other men with their children in the park, I turn my face away. The guilt is too heavy*”

This vivid expression encapsulates the acute emotional distress experienced by migrant fathers who, despite working tirelessly to support their families from abroad, struggle with a profound sense of guilt and loss stemming from their physical absence. This guilt is intensified by the comparison to visibly engaged fathers in the host country. Witnessing their interactions with their children can trigger feelings of failure and exclusion, reinforcing the emotional toll of transnational separation (Chereni, 2015). The impact extended to the home through digital conversations and updates from spouses. A father from Bergamo lamented,

“*My wife tells me the kids ask why I don't come to school events. I tell them I miss them, but inside, I'm breaking*”

This statement reveals the deep emotional rupture caused by physical absence in the intimate rhythms of a child's life. School events, though often seen as mundane, hold profound symbolic value in the construction of parent-child relationships. For migrant fathers, missing these milestones became a recurring reminder of their exclusion from everyday family life and a source of acute emotional pain and identity destabilization. Transnational parenting often relies on digital communication and emotional labor to bridge the gap, but these efforts are inherently limited (Gonzalez and Katz, 2016; Wang and Lim, 2020). This absence is felt not only by the children, who interpret it as emotional distance, but also by fathers themselves, who internalize their inability to be present as personal failure (Schwartz, 2020). This emotional fracture, shaped by structural and cultural forces, exposes the limitations of a caregiving model that reduces fatherhood to breadwinning and occasional voice/video calls, while denying father's fundamental human need to be physically present to witness, support, and be seen by their children in real time.

## Coping through faith and hope

For many migrant fathers, turning to religion was a vital coping mechanism to relieve emotional strain. They used to offer prayers (*salat*) and seek assistance from God (*Allah*) through supplication (*dua*) that provide a sense of solace and hope as religious faith can serve as a powerful source of emotional resilience, offering comfort and a framework for understanding and enduring hardships (Koenig et al., 2012), especially in the context of transnational migration. As one father in Naples shared,

“*I pray that Allah keeps them safe. That's all I can do from here*”

This statement reflects the emotional surrender and spiritual resilience of many Pakistani migrant fathers who grappled with the limits of their physical absence. For these men, prayer became more than a religious obligation. It served as an emotional lifeline and an active form of caregiving across borders. In the context of Islamic belief systems, prayer (Dua) is imbued with meaning as both supplication and trust in divine will (Izzo, 2025), allowing fathers to maintain a sense of agency even when separated from their families. This reliance on faith is intertwined with the belief in future reunification, a hope that provides emotional endurance. A father in Bergamo reiterated,

“*I remind myself this is sacrifice. It's not forever. One day I will return and be the father they deserve*”

This statement encapsulates the future-oriented hope that sustained many Pakistani migrant fathers through prolonged emotional hardship and familial separation. Migration, in this context, is framed not as abandonment but as a meaningful sacrifice, a temporary rupture undertaken to secure a better future for one's family (Dreby, 2010). This forward-looking narrative reaffirms their role as moral agents and loving fathers, even when material conditions and physical separation suggest otherwise. Beyond religion, small rituals like watching old videos of their children, became affective lifelines for migrant fathers. As one father noted,

“*Sometimes, I watch old videos of my children laughing. That gives me strength to go back to work the next day*”

These moments, though technologically mediated, helped fathers reconnect with their caregiving identities and preserve emotional continuity with their children. Such practices served as emotional anchor points, allowing fathers to reaffirm their emotional bonds even in absence (Wang and Lim, 2021). Faith and hope served as active coping tools, helping migrant fathers preserve their identity and endure the strain of prolonged separation.

## Conclusion

This study reveals the deeply emotional and psychologically taxing nature of transnational fatherhood among Pakistani migrants in Italy, where physical separation from children necessitates a renegotiation of traditional paternal roles that emphasize presence, protection, and emotional engagement (Jeong et al., 2023; Kay, 2009). Fathers grapple with profound feelings of guilt, helplessness, and identity conflict as they are reduced to distant providers, often performing emotional labor to mask their distress while coping with the fear of weakening family bonds (Barrett and Charlton, 2025; Carling et al., 2012; Madianou and Miller, 2011). Special occasions and family crises further intensify this emotional chasm, highlighting the limits of digital communication in replicating the intimacy and sensory experience of caregiving (Baldassar and Wilding, 2020; Marchetti-Mercer and Swartz, 2020). Despite these challenges, many fathers find solace in religious faith and the hope of future reunification, which serve as crucial coping mechanisms to sustain emotional resilience and paternal identity (Dreby, 2010; Koenig et al., 2012). These findings call for immigration and family policies that recognize the emotional dimensions of transnational parenting and provide support structures to mitigate the psychological toll on migrant fathers and their families.

## Data Availability

The raw data supporting the conclusions of this article will be made available by the authors, without undue reservation.
